# Influential Attributes on Medical Expense for Korean Older Adults Based on Mental Accounting: Panel Data Analysis Using Korean Longitudinal Study of Aging Data

**DOI:** 10.3390/healthcare13050558

**Published:** 2025-03-05

**Authors:** Min Gyung Kim, Joonho Moon

**Affiliations:** 1 College of Business Management, Hongik University, 2639, Sejong-ro, Jochiwon-eup, Sejong-si 30016, Republic of Korea; mkim@hongik.ac.kr; 2 Department of Tourism Administration, Kangwon National University, Chuncheon 24341, Republic of Korea

**Keywords:** medical expense, housing expense, food expense, Korean older adults, inverted U-shape relationship

## Abstract

**Backgrounds:** Korean society is entering an aging society, and this phenomenon indicates the need for preparation for aging in Korean society. In such a situation, exploring the characteristics of the elderly can be considered important for preparation. The objective of this study is to identify the determinants of medical expenses among older adults in South Korea. The key factors analyzed include food, leisure, and housing expenses, as well as lifestyle choices such as drinking and smoking. **Method**: Data from the Korean Longitudinal Study of Aging, covering 7374 observations from the years 2018 and 2020, are adopted for statistical analysis. This research explores the inverted-U-shape effect of food, leisure, and housing expenditures on medical costs, grounded in the concepts of diminishing marginal utility and mental accounting. A quadratic panel regression analysis is used to test the hypotheses, controlling for variables such as birth year, gender, and personal assets. **Results:** The results show that food and housing expenses have an inverted-U relationship with medical expenditures based on diminishing marginal utility and mental accounting as the theoretical foundation. However, leisure expenses, drinking, and smoking do not significantly affect medical expenses. Furthermore, this study identifies the optimal expenditure levels for maximizing medical spending through the first-order condition. **Conclusions:** These findings provide important insights for the development of policies aimed at improving the financial well-being of older adults in South Korea. Moreover, this study contributes to the literature by applying the concepts of mental accounting and the law of diminishing marginal utility to better understand the financial behavior of older adults.

## 1. Introduction

Choi and Yoo [1] emphasize that medical costs represent a significant financial burden for older adults, who are more susceptible to illness compared to younger populations. Previous studies have highlighted that medical expenses not only contribute to individual financial strain but also to broader social costs, as they are linked to insurance expenditures and reflect the rising costs older adults incur to maintain their health [2,3]. Given these considerations, understanding the factors driving medical expenses among older adults is crucial. Therefore, this study aims to explore the behavioral characteristics associated with medical expenditure in this demographic, with medical expenses serving as the dependent variable.

The determinants of medical expenses identified in this study include food expenses, leisure expenses, housing expenses, and lifestyle factors such as smoking and drinking. Researchers alleged that food expenses are closely related to an individual’s health condition, which in turn influences medical costs [4,5]. Moreover, previous studies stated that leisure activities are recognized as vital for improving both mental and physical health, contributing to a reduction in healthcare needs [6,7]. Prior works also noted that housing quality linked with cost has appeared as a critical factor influencing health because it affects an individual’s recovery and well-being [8,9]. Furthermore, prior research contended that spending patterns are important because they reflect individuals’ behavioral characteristics [10,11]. Hence, they are likely to provide insights into their health-related expenditures. Finally, smoking and drinking, which have been shown to negatively affect health [12,13,14,15], are expected to account for higher medical expenses.

This study adopts the concept of mental accounting, which suggests that individuals categorize and evaluate expenditures across different mental accounts [16,17]. Specifically, this research aims to explore how medical expenses among older adults are influenced by expenditures in other categories, such as food, leisure, and housing. Additionally, this study is grounded in the law of diminishing marginal utility, which posits that the marginal utility of goods decreases as their consumption increases [17]. In this context, spending on food, leisure, and housing is often managed separately, potentially influencing decisions related to medical spending. This study applies this principle to examine the financial behavior of older adults in South Korea, suggesting that excessive consumption of certain goods—such as food and housing—may contribute to financial strain, particularly for older adults [17].

The primary objective of this study is to identify the key determinants of medical expenses among Korean older adults, with a focus on food, leisure, housing expenses, smoking, and drinking. While medical costs are a critical concern for older adults, research on the specific factors influencing these costs remains limited. This study seeks to fill this gap by examining the determinants of medical expenditures in South Korea, a country experiencing rapid demographic aging [18,19]. Thus, this research makes a significant contribution to the literature by integrating the theoretical framework of mental accounting to better understand the behavior of older adults in South Korea. By using mental accounting as a lens, this study aims to uncover the relationships between various expenditure behaviors in older populations. Given the country’s demographic trends, South Korea provides an ideal context for exploring the behavioral dynamics that shape medical expenditures among older adults. The findings of this study are expected to offer valuable insights for policymakers aiming to design welfare programs that promote the financial well-being of the elderly.

## 2. Review of the Literature and Hypothesis Development

### 2.1. Medical Expense

Medical expenses refer to the financial resources allocated for addressing an individual’s health needs [2,20]. For older adults, medical expenses are a significant cost burden, as the likelihood of illness increases with age [1,2,3]. It can be inferred that older adults might need to pay more for the medical service. Choi and Yoo [1] argue that medical expenses contribute significantly to household debt, exacerbating financial strain for older adults, whose incomes are generally more limited compared to younger populations. Tur-Sinai [21] and Chen et al. [3] emphasize that older adults are particularly vulnerable to illness, leading to higher medical expenditures for both preventive care and recovery. This increased spending results from the higher likelihood that illnesses in older adults may lead to severe or fatal outcomes. Moreover, rising life expectancy has made medical costs an increasingly critical factor in financial planning, as these expenses accumulate over a longer lifespan, often surpassing available insurance coverage for older individuals [22,23]. This trend highlights the growing financial burden of medical expenses, especially in the context of extended life expectancies. Fan et al. [24] and Kim and Jacobson [25] further stress the importance of understanding the dynamics of medical expenditures among older adults, given the substantial social costs associated with healthcare. Collectively, the existing literature emphasizes the rising significance of medical expenses as an urgent and escalating issue, particularly for aging populations.

### 2.2. Food and Health Condition

Food is a fundamental necessity for sustaining life, providing the essential energy required for daily functioning [26,27]. The existing literature highlights the importance of food quality in promoting optimal health, as nutritious food plays a key role in enhancing the immune system [4,5]. Accessing higher-quality food often requires individuals to incur greater costs due to its relative scarcity [27,28]. High-quality food is typically characterized by a balanced nutritional profile, including essential macronutrients such as proteins, fats, and carbohydrates [4,27]. In contrast, low-quality food, which is often more affordable, is linked to negative health outcomes such as obesity and diabetes, thereby increasing healthcare demands. Malnutrition, in particular, has a significant inverse relationship with the health and well-being of older adults [29]. Rigling et al. [30] highlight that poor dietary quality among older adults is associated with a higher likelihood of illness, particularly in Switzerland. This is of particular concern given that inexpensive foods often contain harmful ingredients, including additives and excessive cholesterol levels [31,32]. Jeong and Seo [33] further stress the importance of food quality for older adults, noting its critical role in maintaining both mental and physical vitality. Based on these findings, it is reasonable to infer that older adults may prioritize higher-quality food to sustain their health, potentially resulting in increased food-related expenditures. Given these considerations, it is reasonable to infer that older adults may prioritize consuming higher-quality food to maintain better health, potentially leading to increased spending on food. This increased expenditure could, in turn, impact their medical budgets by reducing the resources available for healthcare-related costs.

### 2.3. Leisure and Health Condition

Previous research has emphasized the significant role of leisure activities in promoting better health by reducing stress and stimulating metabolic functions [6,7]. Scholars have argued that leisure activities are integral to fostering healthier aging, as they enhance physical activity, encourage social interaction, and combat isolation, thereby supporting both mental and physical well-being [34,35]. Furthermore, Roy and Orazem [36] and Lee et al. [37] highlight that active leisure is crucial for improving overall health by promoting greater mental engagement. Fancourt et al. [38] also assert that leisure activities contribute to improved health outcomes by allowing individuals to fulfill various needs, such as socializing and participating in physical activities. Numerous studies have demonstrated the positive impact of leisure on health. For instance, Hutchinson and Kleiber [39] found that leisure activities play a crucial role in maintaining health among retirees by reducing the likelihood of social isolation. Similarly, Kim et al. [40] conducted a study on the Korean population and reported a positive association between leisure engagement and improved health, with benefits stemming from both physical and mental stimulation. In a meta-analysis, Yueh and Chang [41] concluded that leisure participation is particularly important for older adults, as it enhances physical strength and reduces the likelihood of isolation, which is closely linked to mental health issues.

### 2.4. Housing and Health Condition

Housing serves as a fundamental shelter, protecting individuals from environmental conditions [42,43]. Beyond providing basic protection, housing plays a critical role in health by offering a space for rest and recovery, particularly in the private setting where individuals spend a significant amount of time [7,8,44]. Previous research suggests that, in later life, individuals tend to adopt less active lifestyles compared to their younger years, resulting in increased time spent at home [44,45]. Bhat et al. [46] conducted a longitudinal study of older adults in the United States, revealing that housing instability has a detrimental effect on health. Poor housing conditions can impede both mental and physical recovery, emphasizing the critical role of housing quality in overall health, as its primary function is to provide comfort. Ahmad et al. [47] found a connection between inadequate housing and increased mortality rates, noting that poor housing conditions elevate the risk of illness and crime. Matte and Jacobs [9] further argue that homelessness in the United States represents a significant public health issue, given the health risks associated with substandard housing, such as unintentional injuries, infections, and indoor air pollution. Similarly, Khodabakhsh [45] underscores the importance of housing for older adults, highlighting that their reduced ability to engage in outdoor activities often leads to spending more time indoors compared to younger individuals.

### 2.5. Expense and Quality Based on Diminishing Marginal Utility with Mental Accounting

Previous research suggests that individuals’ spending patterns reflect their values and beliefs [10,11,48]. Lin and Peng [49] outline three key aspects of the law of diminishing marginal utility: the initial increase in utility, the subsequent decline in additional utility, and its impact on individual decision-making. The literature further highlights a positive relationship between higher-quality goods and increased expenditure, indicating that analyzing expenditure patterns provides valuable insights into individuals’ underlying preferences and priorities [50,51]. Conversely, lower-quality goods and services are typically associated with reduced costs [52,53,54]. Rao [55] argues that price often signals quality, a concept that applies to various goods and services, including food, leisure, and housing. This indicates that lower-cost goods and services may provide limited utility, implying that suboptimal food, leisure, and housing may be insufficient to support good health. The principle of diminishing marginal utility posits that the additional benefit derived from consuming one more unit of a good decreases as consumption increases [56,57]. This principle can be applied to the repetitive consumption of goods such as food, housing, and leisure, where the value or satisfaction derived tends to diminish over time. Consequently, older adults may experience diminishing returns from these types of consumption, potentially leading to a reluctance to allocate their resources toward healthcare spending.

Li and Hsee [58] and Mahapatra et al. [59] further suggest that individuals experience a greater financial burden in one area when excessive expenditure in that area reduces available resources for other areas. This aligns with the concept of mental accounting, as proposed by Henderson and Peterson [60] and Thaler [16], which posits that individuals allocate their money into distinct mental accounts based on the source or intended purpose of the funds. Mental accounting refers to the cognitive process by which individuals categorize, assess, and track their financial activities in separate “accounts” in their minds [59,60]. As a result, spending resources in one category can impact the surplus available for other categories, as individuals often engage in categorical budgeting. Gross et al. [17] explored healthcare consumption behavior by applying mental accounting as a theoretical framework. Building on these insights, it is reasonable to infer an inverted-U-shaped relationship between expenditure and health outcomes, with the effect on medical expenses being particularly pronounced. Based on this framework, the following research hypotheses are proposed:

**Hypothesis 1.**  *Food expense exerts an inverted-U-shape impact on medical expenses.*

**Hypothesis 2.**  *Leisure expense exerts an inverted-U-shape impact on medical expenses.*

**Hypothesis 3.**  *Housing expense exerts an inverted-U-shape impact on medical expenses.*

### 2.6. Smoking, Drinking, and Health

Previous research has consistently shown that smoking is a major contributor to various health issues, including lung cancer and respiratory diseases [15,61,62]. Haghani et al. [12] further demonstrated that smoking habits significantly increase the risk of cancer. The literature also indicates that smoking is linked to an elevated risk of stroke and heart disease [63,64,65]. Similarly, alcohol consumption has been associated with negative effects on liver function and a range of adverse health outcomes, including obesity and high blood pressure [13,66]. These findings imply that both smoking and alcohol consumption are negatively associated with individual health, thereby contributing to higher personal expenditures on medical services. Klatsky and Udaltsova [14] reported that alcohol consumption increases the risk of mortality. Moreover, previous studies have highlighted the association between drinking and an elevated risk of various illnesses, including depression, anxiety, cancer, and cardiovascular disease [67,68,69]. From this literature, it can be inferred that both smoking and drinking are detrimental to health, leading to increased medical expenditures. Based on the literature review, the following research hypotheses are proposed:

**Hypothesis 4.**  *Smoking exerts a positive impact on medical expenses.*

**Hypothesis 5.**  *Drinking exerts a positive impact on medical expenses.*

## 3. Method

### 3.1. Research Model and Data Collection

**Figure 1:**
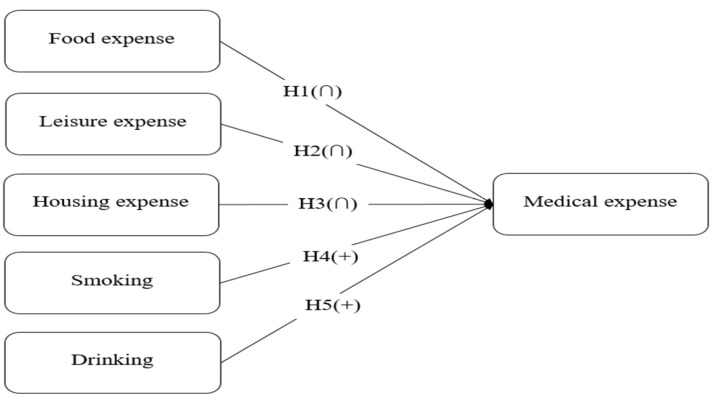
Research model: determinants of medical expense.

Figure 1 is the research model. There are five independent variables: food expense, leisure expense, housing expense, smoking, and drinking. Food expenses, leisure expenses, and housing expenses showed an inverted-U-shaped effect on medical expenses. Smoking and drinking are positively associated with medical expenses.

The data for this study are derived from the Korean Longitudinal Study of Aging (KLoSA), a well-established dataset frequently used in prior research to examine the behaviors and life outcomes of older adults in South Korea [70,71,72]. These studies highlight the reliability and validity of KLoSA data for rigorous statistical analysis. This research utilizes data from two time points: 2018 and 2020. KLoSA offers comprehensive longitudinal data on the social, economic, physical, and mental aspects of older adults’ lives, capturing changes over time that are difficult to observe with cross-sectional surveys—particularly within the context of South Korea’s rapidly aging population. The data are sourced from the Korean Employment Information Service [70,71]. Updated biennially, the most recent data include observations from 2018 and 2020, targeting individuals aged 45 and older [71,72]. As a panel dataset, KLoSA includes repeated observations of the same individuals across these periods, enabling dynamic analysis of individual-level changes over time [73]. In total, the dataset comprises 7374 observations.

### 3.2. Variable Description

Table 1 illustrates the variables used in this study. The survey question regarding medical expenses was phrased as “What is your monthly medical expenditure? (Unit: KRW 10,000)” for respondents to report their expenses. In social welfare budgeting, ensuring the elderly have access to quality food in welfare facilities could reduce costs and improve welfare outcomes, lowering healthcare expenses. The key variables in this study were measured as ratios. To be specific, medical expense (MEX) was measured as the monthly medical costs. Food expense (FEX) was defined as the monthly expenditure on food, while leisure expense (LEX) referred to the monthly spending on leisure activities, and housing expense (HEX) represented the monthly cost of housing. Smoking (SMO) and drinking (DRN) were coded as binary variables based on current status (0 = no, 1 = yes). For example, SMO was measured using the following question: Do you currently smoke? Sex (SEX) was also a binary variable (0 = female, 1 = male). Birth year (BYE) was recorded as the year of birth for each participant, while personal wealth (PWE) was calculated based on the total assets held by the participants.

### 3.3. Data Analysis

For descriptive statistics, this study calculated the mean, standard deviation (SD), minimum, and maximum values. To examine the relationships between the variables, a correlation matrix analysis was performed. For hypothesis testing, this study employed several panel regression models, including ordinary least squares (OLS), fixed effects (FE), and random effects (RE). OLS is a statistical method that minimizes residuals in multiple regression models. The FE model controls for the longitudinal dimension by incorporating year effects as binary variables, while the RE model accounts for unobserved heterogeneity by introducing random components into the regression model [73]. Additionally, instrumental variable (IV) regression analysis was conducted to address potential endogeneity between the dependent and independent variables [73]. This study also examined the potential correlation between the independent variables and the residuals. A significant correlation would suggest the omission of an important variable or a misspecification of the model [73]. However, no statistically significant correlation was found between the independent variables and the residuals. Sensitivity analysis demonstrated the consistency of direction and significance across the three regression models. Furthermore, quadratic regression analysis was conducted, incorporating squared terms to identify the point of maximum effect by applying the first-order condition through differentiation [73]. The squared variables for food, leisure, and housing expenses (FEX, LEX, and HEX) were tested for an inverted-U-shaped relationship with medical expenses. A scatter plot was also provided for graphical representation, allowing for a closer examination of this inverted-U effect. Lastly, an independent *t*-test was performed, and the results were presented using a bar chart to analyze the impact of smoking and drinking on medical expenses.

## 4. Results

### 4.1. Descriptive Statistics and Correlation Matrix

Table 2 presents the descriptive statistics for the variables in this study. The mean value of medical expenses (MEX) is 10.04, with a standard deviation (SD) of 15.07. Food expenses (FEX) have a mean of 43.93 and an SD of 25.56, while leisure expenses (LEX) have a mean of 5.43 and an SD of 9.51. Housing expenses (HEX) show a mean of 15.69 and an SD of 9.43. The descriptive statistics also indicate that 8% of the sample reported smoking, 31% engaged in drinking, and 42% were male. The average birth year (BYE) is 1948.75, and the average personal wealth (PWE) is 22,989.65.

Table 3 is the correlation matrix. MEX positively correlated with FEX (r = 0.077, *p* < 0.05), LEX (r = 0.055, *p* < 0.05), HEX (r = 0.146, *p* < 0.05), and PWE (r = 0.077, *p* < 0.05). FEX positively correlated with LEX (r = 0.300, *p* < 0.05), HEX (r = 0.395, *p* < 0.05), and PWE (r = 0.159, *p* < 0.05). LEX positively correlated with HEX (r = 0.302, *p* < 0.05) and PWE (r = 0.287, *p* < 0.05). HEX positively correlated with PWE (r = 0.197, *p* < 0.05). SMO positively correlated with DRN (r = 0.280, *p* < 0.05).

### 4.2. Results of Hypothis Testing

Table 4 shows the results of hypothesis testing excluding control variables. All three models are statistically significant considering the F-values and Wald χ^2^ (*p* < 0.05). Food expenses (FEX) (β = 0.05, *p* < 0.05) and the squared food expenses (FEX^2^) (β = −3.75 × 10^−4^, *p* < 0.05) significantly affected medical expenses (MED). Additionally, housing expenses (HEX) (β = 0.29, *p* < 0.05) and the squared housing expenses (HEX^2^) (β = −1.25 × 10^−3^, *p* < 0.05) exerted a significant impact on MED. The values from the computation of the first-order condition for FEX and HEX were 66.66 and 116.00, respectively.

Table 5 presents the results of hypothesis testing. All three models are statistically significant, as indicated by the F-values and Wald χ^2^ (*p* < 0.05). Food expenses (FEX) (β = 0.10, *p* < 0.05) and the squared term of food expenses (FEX^2^) (β = −6.18 × 10^−4^, *p* < 0.05) significantly influenced medical expenses (MED). Similarly, housing expenses (HEX) (β = 0.29, *p* < 0.05) and the squared term of housing expenses (HEX^2^) (β = −0.01, *p* < 0.05) had a significant impact on MED. Birth year (BYE) (β = −0.14, *p* < 0.05) and personal wealth (PWE) (β = 2.04 × 10^−5^, *p* < 0.05) were also significant predictors of MED. These results were consistent across all three models, supporting hypotheses H1 and H3. The values of the first-order condition for FEX and HEX were 84.08 and 111.96, respectively.

## 5. Discussion

**Figure 2:**
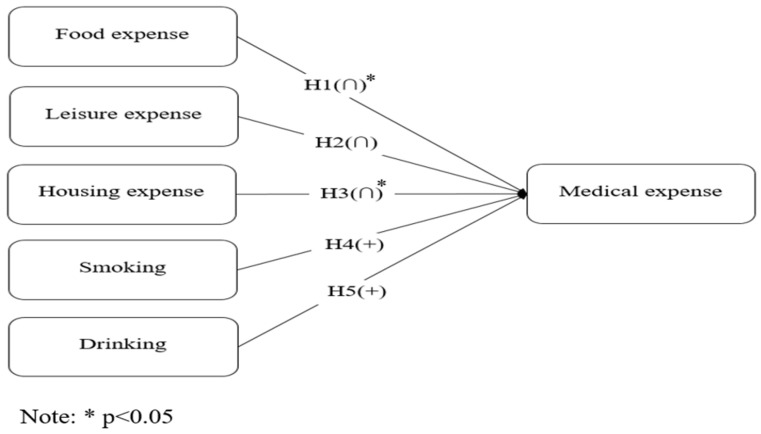
Summary of hypothesis testing results.

This study examines the determinants of medical expenses among older adults in Korea, using data from the KLoSA. With medical expenses as the dependent variable, this study aims to explore the behavioral patterns of older adults in Korea. The analysis is guided by theoretical frameworks based on the law of diminishing marginal utility and mental accounting. This study focuses on data from 2018 and 2020, employing panel regression analysis to generate robust and reliable statistical inferences. Variables such as sex, birth year, and personal wealth are controlled for in the analysis to ensure the accuracy and validity of the results. Figure 2 presents the summary of the hypothesis testing.

**Figure 3:**
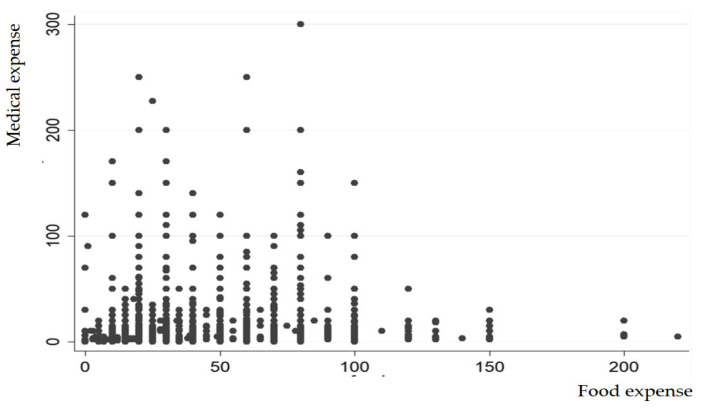
Scatter plot between food and medical expenses.

**Figure 4:**
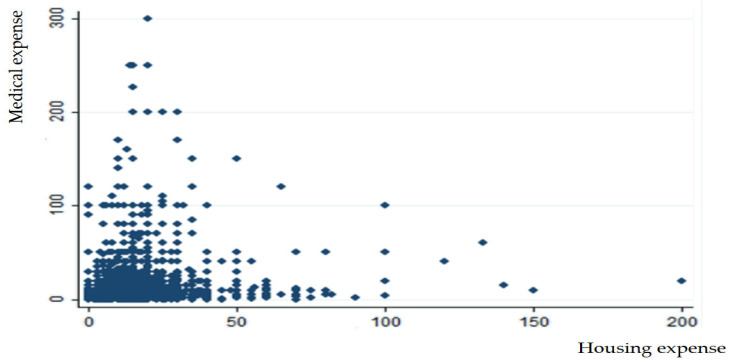
Scatter plot between housing and medical expenses.

The results indicate that both food and housing expenses have an inverted-U-shape effect on medical expenses. The results are explained by the theoretical frameworks of diminishing marginal utility and mental accounting. Figure 3 and Figure 4 present a graphical presentation of the effects of food and housing expenses on medical expense. In detail, the findings indicate that the quality of food and housing plays a significant role in the health of older adults in Korea. Lower expenditures on food and housing may reflect poorer living conditions and diet, which could lead to higher medical costs. In contrast, older adults residing in better housing environments with access to higher-quality food and improved facilities tend to incur lower medical expenses. However, when compared to the expenditure levels that maximize medical costs, the mean values for food and housing expenses were found to be insufficient. In contrast, leisure expenses were found to have no significant impact on medical costs. This may be attributed to the relative importance of basic needs: food and housing are essential for survival, while leisure expenses are more discretionary. Consequently, the lack of a significant relationship between leisure expenditures and medical costs may reflect their secondary role in influencing older adults’ health outcomes. 

**Figure 5:**
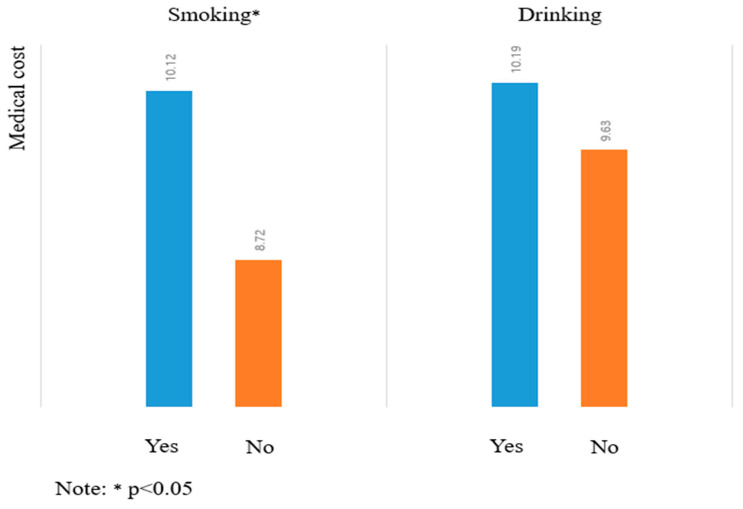
Bar chart presentation based on independent *t*-test in the case of smoking and drinking.

Next, smoking and drinking were identified as non-significant factors in medical expenditure. This finding may be explained by the complex relationship between these behaviors and health. For instance, older adults in relatively good health may be more likely to continue smoking or drinking, or these habits may be deeply ingrained in their lifestyles, making them less likely to directly impact medical costs. The regression analysis further revealed no significant relationship between alcohol consumption, smoking, and medical expenses. It can be inferred that older adults who smoke or drink may not incur higher medical costs, possibly because they are in better health or less concerned about the long-term effects of these behaviors on their health. These findings contrast with studies on Chinese older adults, where smoking and drinking were found to significantly impact medical expenses [74]. This study also found that younger participants tend to allocate less of their budget to medical expenses, whereas wealthier older adults are more likely to spend more on healthcare. This may be because wealthier individuals have greater financial resources, allowing them to allocate funds toward both preventive care and recovery, leading to higher overall medical expenditures. Figure 5 presents the results of the independent *t*-test, showing that only smoking exhibited a significant change. Based on the *t*-test results, no significant difference was found for alcohol consumption. However, it can be inferred that elderly individuals who smoke may be relatively less concerned about their health.

## 6. Conclusions

### 6.1. Theoretical Contributions

This work sheds light on the literature by identifying key determinants of medical expenses among older adults, grounded in the theoretical frameworks of diminishing marginal utility and mental accounting. While medical expenses are a critical concern for the elderly, research on the factors influencing these costs has been relatively limited. Prior studies have documented the substantial financial burden of medical costs on older adults in various contexts, including China, Saudi Arabia, and the United States [75,76,77]. By addressing this gap, this study highlights two essential determinants—food and housing expenses—that significantly influence medical expenditures. The findings underscore the prioritization of essential living expenses for older adults, where food and housing are recognized as fundamental needs, whereas leisure expenses are secondary. In this context, this study demonstrates the explanatory power of mental accounting and diminishing marginal utility in understanding the spending behavior of older adults in South Korea. Specifically, it highlights how expenditures in one “mental account” can influence spending in other accounts, revealing an inverted-U relationship between various types of expenditure (food, housing, and leisure) and medical costs. This represents a significant academic contribution by providing new insights into the dynamics of spending behavior and its implications for healthcare costs among aging populations.

Furthermore, this work academically contributes to the literature by opening a path for exploring the causal relationship between smoking, drinking, and health outcomes in older adults. Two potential scenarios arise from these findings: First, healthier older adults may engage in smoking and drinking without incurring substantial health costs, implying a lifestyle choice rather than a direct health consequence. Alternatively, smoking and drinking may exacerbate health issues in older adults, directly increasing their medical expenditures. This nuanced understanding contributes to the literature by highlighting the distinct effects of alcohol consumption and smoking on the health and financial well-being of older adults.

### 6.2. Policy Implications

The results of this study have important policy implications. Policymakers might be able to consider allocating more resources to support food and housing expenses for older adults. For food, the government could introduce food vouchers or work with food suppliers to control prices for the elderly. In the context of budgeting for social welfare policies, one potential strategy is to ensure that the elderly have access to high-quality food in welfare facilities. Specifically, providing food in large quantities at locations frequently used by older adults could yield significant benefits for elderly welfare at a lower cost. This, in turn, could help reduce healthcare expenses. Similarly, housing subsidies could alleviate the financial burden of housing costs for older adults. Further investment in expanding housing options for the elderly—through both new construction and the renovation of existing properties—would help ease the financial strain associated with food and housing costs, ultimately improving the health and well-being of older adults.

In policy design, this study suggests that policymakers might be able to focus limited resources on supporting essential goods—such as food and housing—rather than discretionary expenses like leisure. Also, as older adults increasingly allocate a larger proportion of their income to healthcare as they age, it is crucial to design welfare policies tailored specifically to this demographic. Investing in enhancing the income of older adults, particularly through employment support programs, would not only improve their financial well-being but also bolster their sense of self-worth and social value. Based on the findings of the current work, it is essential to focus on providing support for food and housing costs to individuals receiving healthcare subsidies. This could involve offering food vouchers or providing affordable housing options, rather than direct cash assistance. Specifically, placing restrictions on how the subsidy can be used would ensure that it is spent on relevant necessities. These considerations should be integrated into the development of healthcare policies designed to address the needs of an aging population.

### 6.3. Limitations and Suggestions for Future Research

Despite its contributions, this research has limitations. First, the sample is limited to older adults in Korea. Future studies could expand the scope by examining diverse national contexts, thereby enhancing the generalizability of the findings. Second, this study identified only two significant factors influencing medical expenses, and the R-squared value was relatively low. Therefore, future research could explore additional variables to provide a more comprehensive understanding of the key determinants of medical expenditures. These efforts will contribute to a deeper understanding of the healthcare needs of older adults and offer valuable insights for developing more effective policies to improve their welfare. Lastly, this research focused on individual variables to explain medical expenses, which may limit the understanding of older adults’ behavior. Scholars could consider incorporating interaction variables to further examine the determinants of medical expenses.

## Figures and Tables

**Table 1:** Depiction of variables.

Variable	Measurement
Medical expense (MEX)	Monthly medical expense
Food expense (FEX)	Monthly food expense
Leisure expense (LEX)	Monthly leisure expense
Housing expense (HEX)	Monthly housing expense
Smoking (SMO)	(0 = no, 1 = yes)
Drinking (DRN)	(0 = no, 1 = yes)
Birth year (BYE)	Birth year of participants
Sex (SEX)	(0 = female, 1 = male)
Personal wealth (PWE)	Personal assets possessed by survey participant

Note: unit of expense and personal wealth (Korean won: KRW).

**Table 2:** Results of descriptive statistics (n = 7374).

Variable	Mean	SD	Minimum	Maximum
MEX	10.04	15.07	0	300
FEX	43.93	25.56	0	220
LEX	5.43	9.51	0	200
HEX	15.69	9.43	0	200
SMO	0.08	0.27	0	1
DRN	0.31	0.46	0	1
BYE	1948.75	9.81	1916	1963
SEX	0.42	0.49	0	1
PWE	22,989.65	36,555.53	−35,000	788,000

Note: SD denotes standard deviation, MEX: medical expense, FEX: food expense, LEX: leisure expense, HEX: housing expense, SMO: smoking, DRN: drinking, BYR: birth year, SEX: sex, and PWE: personal wealth.

**Table 3:** Results of the correlation matrix.

Variable	1	2	3	4	5	6	7	8
1. MEX	1							
2. FEX	0.077 *	1						
3. LEX	0.055 *	0.300 *	1					
4. HEX	0.146 *	0.395 *	0.302 *	1				
5. SMO	−0.022 *	0.020 *	0.024 *	0.031 *	1			
6. DRN	−0.016	0.135 *	0.119 *	0.079 *	0.280 *	1		
7. BYE	−0.030 *	0.369 *	0.265 *	0.250 *	0.120 *	0.271 *	1	
8. SEX	0.012	0.037 *	0.042 *	0.046 *	0.308 *	0.379 *	0.032 *	1
9. PWE	0.077 *	0.159 *	0.287 *	0.197 *	0-.003	0.089 *	0.069 *	0.162 *

Note: * *p* < 0.05, MEX: medical expense, FEX: food expense, LEX: leisure expense, HEX: housing expense, SMO: smoking, DRN: drinking, BYR: birth year, SEX: sex, and PWE: personal wealth.

**Table 4:** Results of hypothesis testing excluding control variables.

Variable	Model 1 (OLS) β (t-Value)	Model 2 (FE) β (t-Value)	Model 3 (RE) β (Wald)	Model 4 (IV) β (t-Value)
Intercept	4.63 (8.60) *	4.63 (8.60) *	4.63 (8.60) *	4.63 (8.60) *
FEX	0.05 (2.84) *	0.05 (2.84) *	0.05 (2.84) *	0.05 (2.84) *
FEX2	−3.75 × 10^−4^ (−2.47) *	−3.75 × 10^−4^ (−2.47) *	−3.75 × 10^−4^ (−2.47) *	−3.75 × 10^−4^ (−2.47) *
LEX	0.01 (−0.18)	0.01 (−0.18)	0.01 (−0.18)	0.01 (−0.18)
LEX2	−1.77 × 10^−4^ (0.58)	−1.77 × 10^−4^ (0.58)	−1.77 × 10^−4^ (0.58)	−1.77 × 10^−4^ (0.58)
HEX	0.29 (9.65) *	0.29 (9.65) *	0.29 (9.65) *	0.29 (9.65) *
HEX2	−1.25 × 10^−3^ (−3.52) *	−1.25 × 10^−3^ (−3.52) *	−1.25 × 10^−3^ (−3.52) *	−1.25 × 10^−3^ (−3.52) *
SMO	−1.31 (−1.90)	−1.31 (−1.90)	−1.31 (−1.90)	−1.31 (−1.90)
DRN	−2.59 (−2.59) *	−2.59 (−2.59) *	−2.59 (−2.59) *	−2.59 (−2.59) *
F-value	28.41 *	28.41 *	-	28.41 *
Wald χ2	-		227.26 *	-
R2	0.0257	0.0257	0.0320	0.0257

Note: * *p* < 0.05, MEX: medical expense (dependent variable), FEX: food expense, LEX: leisure expense, HEX: housing expense, SMO: smoking, DRN: drinking, Δ/ΔFEX = 66.66, and Δ/ΔHEX = 116.00.

**Table 5:** Results of hypothesis testing including control variable.

Variable	Model 5 (OLS) β (t-Value)	Model 6 (FE) β (t-Value)	Model 7 (RE) β (Wald)	Model 8 (IV) β (t-Value)
Intercept	279.23 (6.94) *	279.23 (6.94) *	279.23 (6.94) *	279.23 (6.94) *
FEX	0.10 (4.64) *	0.10 (4.64) *	0.10 (4.64) *	0.10 (4.64) *
FEX2	−6.18 × 10^−4^ (−3.80) *	−6.18 × 10^−4^ (−3.80) *	−6.18 × 10^−4^ (−3.80) *	−6.18 × 10^−4^ (−3.80) *
LEX	0.01 (0.63)	0.01 (0.63)	0.01 (0.63)	0.01 (0.63)
LEX2	−9.41 × 10^−5^ (−0.30)	−9.41 × 10^−5^ (−0.30)	−9.41 × 10^−5^ (−0.30)	−9.41 × 10^−5^ (−0.30)
HEX	0.29 (8.54) *	0.29 (8.54) *	0.29 (8.54) *	0.29 (8.54) *
HEX2	−1.36 × 10^−3^ (−3.68) *	−1.36 × 10^−3^ (−3.68) *	−1.36 × 10^−3^ (−3.68) *	−1.36 × 10^−3^ (−3.68) *
SMO	−1.07 (−1.42)	−1.07 (−1.42)	−1.07 (−1.42)	−1.07 (−1.42)
DRN	−0.65 (−1.52)	−0.65 (−1.52)	−0.65 (−1.52)	−0.65 (−1.52)
BYE	−0.14 (−6.83) *	−0.14 (−6.83) *	−0.14 (−6.83) *	−0.14 (−6.83) *
SEX	0.16 (0.38)	0.16 (0.38)	0.16 (0.38)	0.16 (0.38)
PWE	2.04 × 10^−5^ (3.74) *	2.04 × 10^−5^ (3.74) *	2.04 × 10^−5^ (3.74) *	2.04 × 10^−5^ (3.74) *
F-value	22.11 *	20.27 *	-	22.11 *
Wald χ2	-	-	243.22 *	-
R2	0.0320	0.0320	0.0320	0.0320

Note: * *p* < 0.05, MEX: medical expense (dependent variable), FEX: food expense, LEX: leisure expense, HEX: housing expense, SMO: smoking, DRN: drinking, BYR: birth year, SEX: sex, PWE: personal wealth, Δ/ΔFEX = 84.08, and Δ/ΔHEX = 111.96.

## Data Availability

The data presented in this study are available upon request from the corresponding author. The data are not publicly available due to privacy concerns.
